# The DUSP–Ubl domain of USP4 enhances its catalytic efficiency by promoting ubiquitin exchange

**DOI:** 10.1038/ncomms6399

**Published:** 2014-11-18

**Authors:** Marcello Clerici, Mark P. A. Luna-Vargas, Alex C. Faesen, Titia K. Sixma

**Affiliations:** 1 Department of Biochemistry, The Netherlands Cancer Institute, Plesmanlaan 121, 1066CX Amsterdam, The Netherlands

## Abstract

Ubiquitin-specific protease USP4 is emerging as an important regulator of cellular pathways, including the TGF-β response, NF-κB signalling and splicing, with possible roles in cancer. Here we show that USP4 has its catalytic triad arranged in a productive conformation. Nevertheless, it requires its N-terminal DUSP–Ubl domain to achieve full catalytic turnover. Pre-steady-state kinetics measurements reveal that USP4 catalytic domain activity is strongly inhibited by slow dissociation of ubiquitin after substrate hydrolysis. The DUSP–Ubl domain is able to enhance ubiquitin dissociation, hence promoting efficient turnover. In a mechanism that requires all USP4 domains, binding of the DUSP–Ubl domain promotes a change of a switching loop near the active site. This ‘allosteric regulation of product discharge’ provides a novel way of regulating deubiquitinating enzymes that may have relevance for other enzyme classes.

## Abstract (web summary)

Ubiquitin-specific protease USP4 regulates several cellular signalling pathways. Here, Clerici *et al.* show that the DUSP–Ubl domain of USP4 is required for full catalytic activity, by enhancing the release of ubiquitin from the catalytic site after substrate hydrolysis.

## Graphical abstract

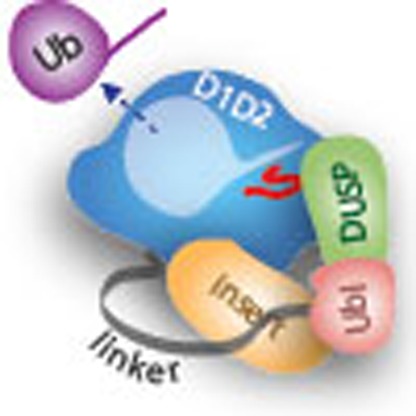

The crucial role of ubiquitin signalling in numerous pathways that control fundamental aspects of cellular life requires the fine balance between the action of ubiquitin-conjugating and -deconjugating enzymes. Therefore, these undergo a tight regulation at the cellular level, by controlling enzyme abundance and localization, but also at the protein level by evolving specificity in substrate recognition and the ability to modulate their catalytic activity.

Ubiquitin-specific proteases (USPs) represent the largest family of deubiquitinating enzymes (DUBs) counting >60 members in humans. The catalytic domain of USPs shows a conserved fold with papain-like architecture and an extended finger-like region that forms the ubiquitin-binding pocket^1^. In addition, each USP carries a unique modular combination of other domains located N-terminally, C-terminally or within the catalytic core in preferred integration sites^2^. These domains perform a variety of tasks including substrate recognition, recruitment of regulatory factors and subcellular localization. The considerable variability resulting from this multi-domain architecture is believed to play a crucial role in the ability of USPs to carry out specific functions^3^.

The domains that are most frequently represented in USPs include zinc-finger domains (ZnF), ubiquitin-like domains (Ubl)^4^ and DUSP domains (domain in USP)^3^. ZnF domains can recognize the ubiquitin C-terminal diglycine motif and function as sensors of the free ubiquitin pool^5^. Ubl domains share the conserved β-grasp fold of ubiquitin, but lack the final GG residues and frequently have evolved to different functions^6^,^7^,^8^. DUSP domains have no described function and are found exclusively in USPs^3^.

Often, the full catalytic competence of a USP enzyme does not rely solely on the catalytic domain but requires the action of its additional domains, the interaction with non-substrate partners or the interplay between the two^9^,^10^,^11^,^12^,^13^. The yeast USP Ubp8 is integrated in a four-subunit machinery (the SAGA DUB module) assembled around the Ubp8 ZnF–UBP domain. Only in this configuration Ubp8 has a competent DUB catalytic centre supported by the Sgf11 subunit packing close to the active site and working as an allosteric regulator^14^,^15^. Analogously, the active site of the USP7 catalytic domain is in a catalytically incompetent conformation^16^ and requires the two C-terminal Ubl domains for full activity. These bind to a specific loop in the catalytic domain (named ‘switching loop' (SL)^8^), promoting rearrangement towards an active conformation; the recruitment of the regulatory protein GMP synthase potentiates this mechanism allosterically, further enhancing USP7 activity^8^.

USP4 is involved in different cellular pathways and targets a variety of substrates. In a mass spectrometry study, USP4 showed extensive interaction with messenger RNA-processing factors, in particular splicing factors^17^. In agreement with this, USP4 has been shown to directly interact with SART3 at the spliceosome where its activity is required for the maturation of the splicing machinery^18^. USP4 also targets ARF-BP1, inhibiting p53-mediated apoptosis and cell cycle checkpoints, and displays elevated levels in several cancer types, making it a potential oncogene^19^. USP4 shuttles between the nucleus and the cytoplasm in an AKT phosphorylation-dependent manner^20^,^21^. At the plasma membrane, it enhances transforming growth factor (TGF)-β response by deubiquitinating and stabilizing the TGF-β type I receptor^20^. USP4 has also been shown to target TRAF2 and TRAF6 inhibiting nuclear factor-κB (NF-kB) signalling and antagonizing lung cancer cell migration^22^,^23^,^24^, suggesting a context-dependent role in cancer.

USP4 features a large domain (~30 kDa, named USP4 insert from here on) inserted within the catalytic core, which carries a Ubl domain. A tandem DUSP–Ubl domain^25^,^26^ is located at the N-terminus of the protein and is separated from the catalytic domain by a connecting linker (Fig. 1a). USP4 shares high sequence similarity and the same domain architecture with two closely related paralogues, USP15 (57% identity) and USP11 (46% identity). Despite high similarity, the targets of the three proteins described so far are mostly non-overlapping.

In this study we show that USP4 requires the N-terminal DUSP–Ubl domain to achieve its full catalytic turnover *in vitro*. USP4 catalytic domain strongly retains ubiquitin after substrate hydrolysis preventing the access of more substrate, thus limiting catalytic turnover. This mechanism involves the SL in the ubiquitin tail-binding cleft. The N-terminal DUSP–Ubl domain is recruited to the catalytic domain by binding to USP4 insert and is able to oppose ubiquitin retention by SL, restoring an efficient catalytic turnover.

## Results

### D1D2 is the minimal catalytic domain of USP4

**Figure 1:**
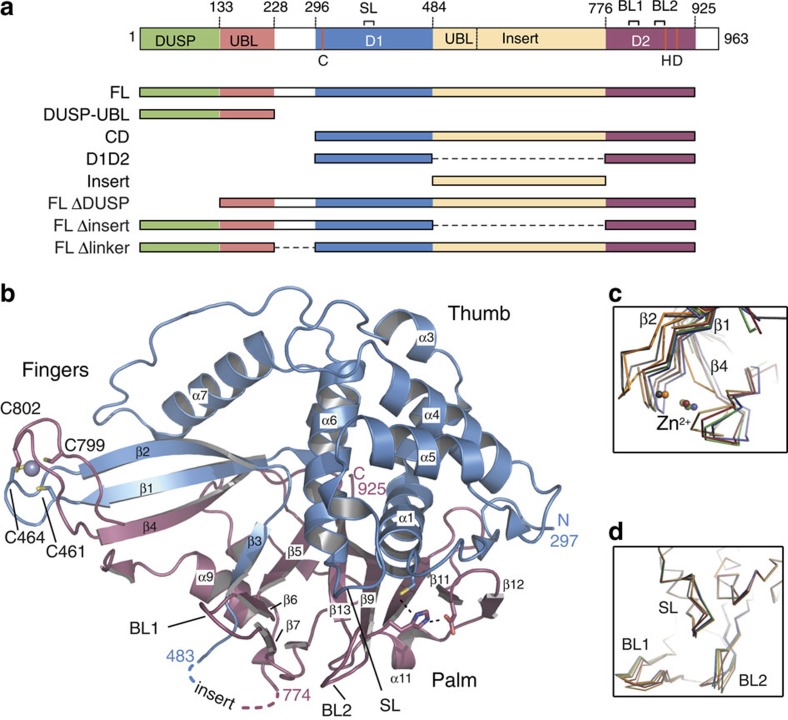
The USP domain of USP4 is formed by the D1 and D2 domains. (**a**) Domain representation of human USP4 and constructs used in this study. The position of the catalytic residues (C, H, D) is indicated in red. SL=switching loop (residues 385–392); BL1 and BL2=blocking loops 1 and 2 (residues 831–834 and 874–880, respectively) (**b**) Cartoon representation of USP4 D1D2 domain crystal structure coloured as in **a**. The catalytic triad and zinc-coordinating residues are represented as sticks. Blocking loops 1 and 2 and switching loop are indicated as BL1, BL2 and SL, respectively. Dotted lines indicate the position of the USP4 insert. (**c**,**d**) Zoom on the superposition of the six non-crystallographic symmetry-related USP4 copies in the asymmetric unit showing the flexibility in the zinc-finger ribbon (**c**) and in the BL1, BL2 and SL loops (**d**).

To gain insight into the structure and function of USP4, we expressed and purified a construct spanning the USP4 catalytic domain (residues 296–954) from *Escherichia coli* and used limited proteolysis to remove disordered regions in the protein in aid of crystallization. After treatment with thermolysin, two fragments—domain 1 (D1) and 2 (D2)—were obtained that copurified on size-exclusion chromatography (Supplementary Fig. 1A,B). We identified the sequence of D1 and D2 using mass spectrometry and N-terminal sequencing (Supplementary Fig. 1C). On the basis of a multi-sequence alignment of USP family members (Supplementary Fig. 1G), we concluded that the protease treatment removed an insertion between L481 and L766 (USP4 insert, Fig. 1a). The two fragments were coexpressed and purified yielding a minimal catalytic domain (USP4 D1D2, Fig. 1a). The split domain retains catalytic function on the minimal substrate ubiquitin–rhodamine 110-glycine (Ub–Rhod)^27^ (Supplementary Fig. 1D).

### Crystal structure of USP4 D1D2

We crystallized and determined the structure of USP4 D1D2 by molecular replacement using the USP8 catalytic domain (PDB 2GFO) as a search model. The structure was refined to 2.4 Å resolution with an *R*/*R*_free_ of 0.178/0.21 and good geometry (Fig. 1b; Supplementary Fig. 1E; Table 1). There are six molecules of USP4 D1D2 per asymmetric unit with equivalent conformation (root mean squared deviation of 0.7 Å over 344 Cαs, calculated with SSM software^28^).

The fold of USP4 D1D2 is similar to that of other USP catalytic domains, resembling an extended right hand comprising three domains: fingers, thumb and palm (Fig. 1b). The D1 fragment contains the thumb domain and part of the fingers domain including the USP signature conserved sequences ‘Cys box’ and ‘QQD box’^29^ of the active site (Supplementary Fig. 1G), whereas the D2 fragment completes the active site with the ‘His box’^29^ and the rest of the fingers and palm domains (Supplementary Fig. 1G). Like other USP apo-structures^30^,^31^,^32^, with the exception of USP7 (ref. 16), the catalytic triad is in a catalytically competent configuration (Fig. 1b).

In the finger domain, D1 and D2 are brought together by a Zn^2+^ ion coordinated by cysteine residues belonging to both domains (Fig. 1b,c). This zinc-finger ribbon (observed also in USP2, USP8 and USP21 structures) is in the contracted ‘closed-hand' configuration seen in USP8, which was proposed to block ubiquitin access^30^. Superposition of the six non-crystallographic symmetry-related molecules of USP4 D1D2 shows flexibility in the zinc-finger ribbon (maximal Cα displacement 4 Å, Fig. 1c), suggesting that it can move to accommodate ubiquitin.

Similarly, three surface loops adopt a variable conformation in the six USP4 copies building the asymmetric unit (Fig. 1d). Blocking loops 1 and 2 (BL1 and BL2) were proposed to block the active site of USP14 and relocate after Ub binding^16^; the third loop corresponds to the SL involved in USP7 activation^8^. In USP4 the position of BL1, BL2 and SL hinders the access of ubiquitin to the catalytic domain (Supplementary Fig. 1F), suggesting that the flexibility observed in these loops is required for the dynamic exchange of ubiquitin in and out of the active site.

The USP4 insert is located between beta-strand β3 (D1) and helix α9 (D2), with its N- and C termini in close proximity (Fig. 1b). To gain information on the position of the insert relative to D1D2, we performed small-angle X-ray scattering (SAXS) on USP4 catalytic domain (CD) (residues 296–925, Fig. 1a) in complex with the suicide inhibitor ubiquitin-propargyl (Ub-Prg)^33^. For comparison we also performed SAXS on the D1D2–UbPrg complex and the isolated insert (residues 483–765, Fig. 1a) (Supplementary Fig. 2A,B). D1D2–UbPrg has a symmetrical distance probability distribution (Supplementary Fig. 2C) in agreement with the globular shape of D1D2, when ubiquitin is modelled in its binding pocket (Supplementary Fig. 2D). The ‘closed-hand’ conformation of D1D2 makes the finger region clash with ubiquitin in the model. Accordingly, the calculated D1D2-Ub-scattering curve fits the experimental data only partially (Supplementary Fig. 2A) and shows a smaller radius of gyration (model *R*_g_=22.9 Å, measured *R*_g_=25.9 Å, supplementary Fig. 2B) than the experimental curve. In contrast to D1D2, the USP4 insert shows an asymmetric distribution indicating that this domain has an elongated shape (Supplementary Fig. 2C). Interestingly, the complete USP4 CD–UbPrg distance distribution function has a similar maximum to D1D2–UbPrg (~36 Å versus ~30 Å) but a significant proportion of additional long distances (Supplementary Fig. 2C), suggesting that the USP4 insert extends from the D1D2 domain into the solvent.

### The DUSP–Ubl domain is required for USP4 activity *in vitro*

**Figure 2:**
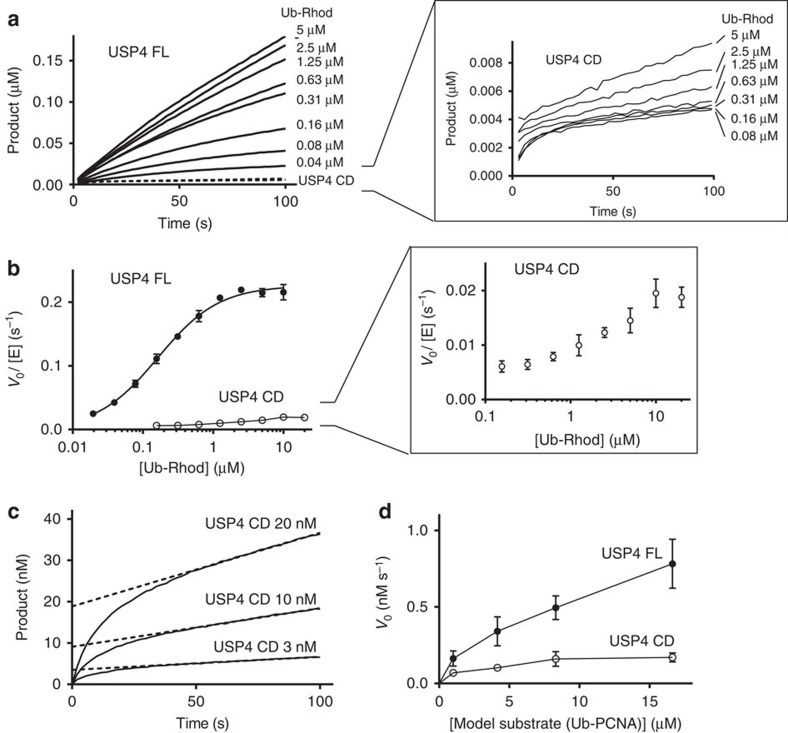
The activity of USP4 is significantly reduced in the absence of the DUSP–Ubl domain. (**a**) Product accumulation in time for USP4 FL and CD reactions on ubiquitin–rhodamine (Ub–Rhod). The panel on the right zooms on USP4 CD curves. (**b**) Michaelis–Menten plots showing the catalytic rate of USP4 FL and CD as a function of substrate concentration (error bars represent s.e.m. for at least three independent replicates). The panel on the right zooms on the USP4 CD curve. (**c**) Product accumulation for USP4 CD reaction at three different enzyme concentrations and 150 nM Ub–Rhod showing that the amplitude of the ‘burst’ phase is equal to the enzyme concentration. (**d**) Michaelis–Menten plot for USP4 FL and CD reaction on the model substrate-ubiquitinated PCNA (error bars represent s.e.m. for at least two independent replicates).

We set out to verify whether the USP4 catalytic domain alone is sufficient to account for the full activity of the enzyme or whether additional domains of the protein are required, as observed for other USPs^9^,^12^,^13^. To this end, we designed two constructs spanning the catalytic domain (USP4 CD, residues 296–925) and the full protein (USP4 FL, residues 8–925), respectively (Fig. 1a), and compared their activity against the minimal substrate Ub–Rhod. USP4 CD has a weak activity compared with the robust activity of USP4 FL (Fig. 2a,b), indicating that the DUSP–Ubl domain is required for full catalytic efficiency of USP4 *in vitro*. To further verify the role of the DUSP–Ubl domain, we performed a Ub–Rhod activity assay where the DUSP–Ubl domain (residues 8–228, Fig. 1a) was added *in trans* to USP4 CD. First, we showed that DUSP–Ubl does not possess DUB activity on Ub–Rhod (Supplementary Fig. 3A), then we mixed it at different concentrations (up to 150 μM) to USP4 CD (50 nM) and added it to the substrate (5 μM). USP4 CD catalytic rates increase up to approximately ninefold for DUSP–Ubl at 150 μM (Supplementary Fig. 3A). This confirms that the catalytically inactive DUSP–Ubl domain can improve USP4 catalytic efficiency also when it is not part of the same protein chain.

The linear phase of USP4 CD product accumulation is preceded by a faster ‘burst’ phase, which is not observed for USP4 FL (Fig. 2a). The amplitude of the ‘burst’ is equivalent to the amount of USP4 CD used in the reaction (Fig. 2c), indicating that USP4 CD is progressively inhibited during the first enzyme turnover before reaching steady-state levels (indicated by the linear accumulation of product). This suggests the possibility that a product of the reaction is retained by USP4 CD and released at a slow rate, preventing the access of more substrate and thus reducing the enzyme turnover. In full-length USP4, Michaelis–Menten parameters can be derived (*k*_cat_=0.30 s^−1^, *K*_M_=0.15 μM, Fig. 2b; Supplementary Fig. 3B; Table 2), but USP4 CD steady-state rates do not fit the Michaelis–Menten model due to the slow product release (Supplementary Fig. 3B). This makes direct comparison of the catalytic parameters of the two enzymes impossible, but the maximum turnover reached by USP4 CD (~0.02 s^−1^ between 10 and 20 μM substrate for USP4 CD) is ~15-fold lower than USP4 FL *k*_cat_, with an apparent *K*_M_ that is higher (half of the maximum turnover is reached at ~1.25 μM substrate) (Fig. 2b). Interestingly, USP4 D1D2 does not show a similar enzymatic behaviour to USP4 CD (Supplementary Fig. 1D; Table 2), suggesting a role of the insert in ubiquitin retention.

We verified that the difference in activity between USP4 catalytic domain and full protein is retained on a substrate where ubiquitin is conjugated to a large protein and is not exclusively relevant for the minimal substrate. As a model protein substrate, we chose recombinant ubiquitinated proliferating cell nuclear antigen (PCNA), which is not among the USP4 described substrates, but it is easy to produce in large amounts^34^. We mono-ubiquitinated PCNA with N-terminally TAMRA-labelled ubiquitin using UbcH5C S22R mutant^34^ and followed deubiquitination in gel assays (Supplementary Fig. 3C). USP4 FL displays significantly higher activity than USP4 CD also on this model substrate (Fig. 2d), confirming that our observations are not limited to minimal substrates.

### USP4 DUSP–Ubl promotes ubiquitin release

**Figure 3:**
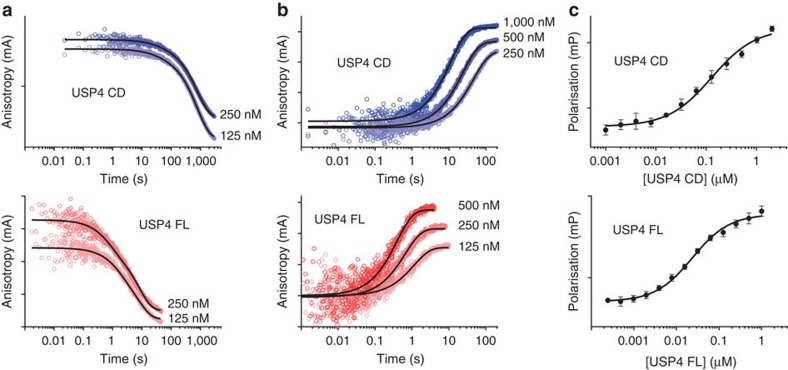
The DUSP–Ubl domain promotes ubiquitin dissociation and association. (**a**,**b**) Pre-steady-state kinetics of ubiquitin dissociation (**a**) and association (**b**) for USP4 CD (upper panel) and FL (lower panel) measured by fluorescence polarization in a stopped-flow device. (**c**) Equilibrium binding of ubiquitin to USP4 CD (upper panel) and FL (lower panel) measured by fluorescence polarization (error bars represent s.e.m. for at least three independent replicates).

Since the low activity observed for USP4 CD suggests a slow product release relative to USP4 FL, we directly measured the kinetics of USP4–ubiquitin dissociation. We used a fluorescence polarization (FP) assay with N-terminally TAMRA-labelled ubiquitin performed in a stopped-flow device that allows fast measurements of pre-steady-state events. USP4 CD and FL were pre-incubated with nanomolar amounts of ^TAMRA^Ub and rapidly mixed with a large excess of non-labelled ubiquitin to avoid rebinding. Whereas ubiquitin release by USP4 FL is completed in <1 min, USP4 CD requires nearly 1 h for full dissociation (Fig. 3a), confirming that the DUSP–Ubl domain activates USP4 by promoting ubiquitin release. Both CD and FL dissociation kinetics are characterized by a slow and a fast component, but in the full-length protein both components are accelerated ~40 times and additionally the fraction of the fast component is much larger (approximately fivefold) (Table 2).

Since ubiquitin release is the limiting step in USP4 CD turnover, the turnover rate should approximate the ubiquitin off-rate. Interestingly, the slowest and major component of ubiquitin dissociation is more than 10-fold slower than the catalytic turnover, suggesting that the energy liberated during ubiquitin hydrolysis may be promoting its dissociation.

When increasing concentrations of the DUSP–Ubl domain were added *in trans* to USP4 CD, an increase in dissociation of ubiquitin was observed in the stopped-flow measurements (Supplementary Fig. 4A). Since the isolated DUSP–Ubl is not able to bind ubiquitin (Supplementary Fig. 4B) this suggests that it acts by rearrangements in the USP4 catalytic domain.

The dissociation constant at equilibrium (*K*_D_) for USP4 FL and CD was also measured with a FP assay. Despite the significant differences in off-rates, the two constructs show similar equilibrium affinities (FL *K*_D_=0.092 μM; CD *K*_D_=0.044 μM, Fig. 3c; Table 2), indicating that DUSP–Ubl promotes also faster on-rates. Indeed, ubiquitin on-rates measurement using FP in the stopped-flow assay show slower association for USP4 CD (*K*_on_=0.074 μM^−1^ s^−1^) relative to USP4 FL (*K*_on_=5.1 μM^−1^ s^−1^) (Fig. 3b; Table 2). Overall, this indicates that the DUSP–Ubl domain has a global effect on the dynamics of ubiquitin exchange by USP4 catalytic domain.

### USP4 requires all domains for its activation

**Figure 4:**
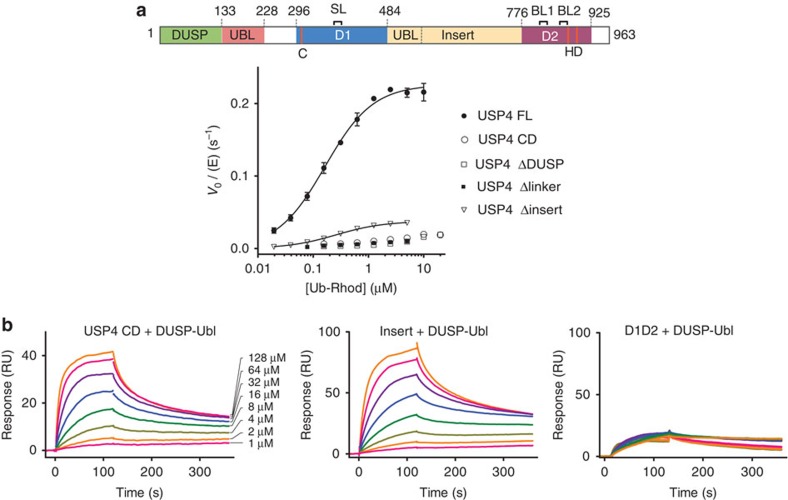
The DUSP domain, the linker and the insert are required for USP4 activity. (**a**) Michaelis–Menten plots showing the catalytic rate of USP4 FL, CD, ΔDUSP, Δlinker and Δinsert as a function of substrate concentration (error bars represent s.e.m. for at least three independent replicates). The domain representation of USP4 indicating the DUSP domain, the insert and the linker is given as reference. (**b**) Surface plasmon resonance (SPR) traces of DUSP–Ubl at different concentrations binding to USP4 CD, insert and D1D2. The overlay of the normalized responses is given in the lower right panel.

To test which regions of USP4 are required for the activation by the DUSP–Ubl domain, we first deleted the DUSP domain from USP4 FL (USP4 ΔDUSP, residues 132–925, Fig. 1a) and analysed the effect of the deletion in a Ub–Rhod assay. USP4 ΔDUSP has substantially lower activity than USP4 FL and is comparable to USP4 CD (Fig. 4a), indicating that the DUSP domain is necessary for the activation of USP4.

Next, we tested the importance of the ~70 residue linker that separates the DUSP–Ubl to the catalytic domain (residues 229–295, Fig. 1a; Supplementary Fig. 4C) and that is predicted to be mostly disordered (analysed with PrDos^35^). When the linker was completely removed (USP4 Δlinker, residues 8–925, deletion of residues 228–296, Fig. 1a), the activity was reduced to the level of USP4 CD (Fig. 4a), indicating that this linker is required for USP4 activation. Since USP4 CD, lacking the linker, can be activated with the addition of DUSP–Ubl *in trans* (Supplementary Fig. 3A), the specific residues may be unimportant, but the flexible linker may rather be required to provide the necessary conformational freedom between DUSP–Ubl and the catalytic domain to allow their interaction and USP4 activation. Since the linker is shortened to ~20 residues in an alternative isoform of USP4 (Supplementary Fig. 3C), we tested the effect of this natural variation on activity. Isoform 2 shows comparable activity to isoform 1 on Ub–Rhod (Supplementary Fig. 3D), indicating that a shorter linker is still compatible with USP4 activation by the DUSP–Ubl domain.

Finally, the role of USP4 insert was investigated by testing the effect of its deletion (USP4 Δinsert, residues 8–925, deletion of residues 485–775, Fig. 1a) on Ub–Rhod hydrolysis. The deletion of the insert significantly reduces the activity of USP4 FL (approximately sevenfold reduction in *k*_cat_, Fig. 4a; Table 2). The inability of DUSP–Ubl to enhance D1D2 activity *in trans* further confirms the importance of the insert in activation (Supplementary Fig. 4E).

### The DUSP Ubl domain interacts with the insert

The activation of USP4 catalytic domain by DUSP–Ubl implies a direct interaction between the two domains. Indeed, surface plasmon resonance (SPR) shows an interaction of DUSP–Ubl with immobilized USP4 CD (Fig. 4b). The measurements were affected by nonspecific interactions of DUSP–Ubl to the SPR chip visible as slow, nonspecific off-rate components. When we excluded these from the calculation using the program EvilFit^36^,^37^, we found apparent *K*_D_ values, *K*_Dfit_ ~100 μM (Supplementary Fig. 4F). This value agrees well with the concentrations required for transactivation (Supplementary Fig. 3A). The SPR measurements also show that DUSP–Ubl is able to bind to the insert alone with similar affinity relative to USP4 CD (*K*_Dfit_ ~44 μM) (Fig. 4b; Supplementary Fig. 4F), indicating that the insert is the main binding region for DUSP–Ubl on the catalytic domain. As a control, USP4 D1D2 does not show specific binding to DUSP–Ubl (Fig. 4b).

As our SAXS data revealed that the insert significantly increases USP4 CD–UbPrg radius of gyration (~70% increase) relative to D1D2–UbPrg (Supplementary Fig. 2B,C), this elongated domain protruding from D1D2 may serve as binding platform for DUSP–Ubl. In contrast, the DUSP–Ubl domain in USP4 FL–UbPrg, induces only a small increase (<10%) in its radius of gyration (*R*_g_=46.9 Å) and size distribution function relative to USP4 CD–UbPrg (*R*_g_=43.9 Å) (Supplementary Fig. 2B,C). This indicates that USP4 FL has a compact arrangement in solution, supporting the conclusion that the DUSP–Ubl domain packs against USP4 CD.

### Paralogues USP11 and USP15 have different modes of regulation

USP4 has two paralogues, USP11 and USP15, with similar domain structure and conserved sequence (Supplementary Fig. 5A). This raises the question whether these paralogues have comparable enzymatic behaviour. Interestingly, removal of DUSP–Ubl in USP15 results in approximately threefold lower activity relative to the full protein, and USP15 CD shows a ‘burst’ phase although less pronounced than in USP4 CD (Table 2; Supplementary Fig. 5B). Thus, USP15 resembles USP4 in its catalytic behaviour, although ubiquitin dissociation does not seem to be as important as in USP4 in affecting the catalytic activity. In USP11 the poor solubility of the CD fragment prevents reliable activity measurements. However, USP11 FL features a ‘burst’ phase (Supplementary Fig. 5C), indicating that, in contrast to USP4 FL and USP15 FL, slow ubiquitin off-rates limit USP11 activity but the DUSP–Ubl domain is not able to promote ubiquitin release or is not efficient in doing so, in agreement with a recent publication^38^.

### Mutations in the DUSP domain reduce USP4 activity

**Figure 5:**
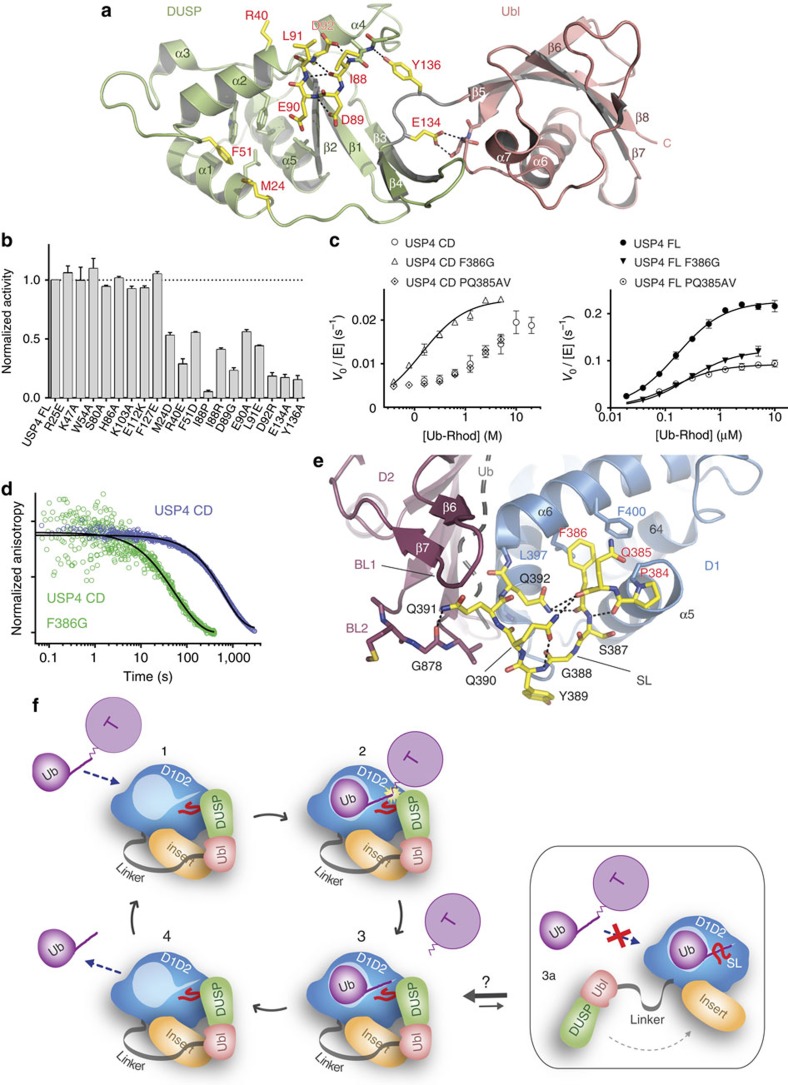
Single-residue mutations in the DUSP domain and in the switching loop hamper USP4 activation. (**a**) Mutations mapped onto structure of the murine DUSP–Ubl domain (PDB 3JYU). Mutated residues that cause a reduction in USP4 FL activity are depicted as yellow sticks and labelled in red. Hydrogen bonds are indicated as black dotted lines. (**b**) Normalized activities of USP4 FL DUSP mutants measured at 5 μM Ub–Rhod concentration (error bars represent s.e.m. for at least three independent replicates). (**c**) Michaelis–Menten plots showing the catalytic rate of F386G and PQ385AV mutants in comparison with USP4 CD (left panel) and USP4 FL (right panel) wild type (error bars represent s.e.m. for at least three independent replicates). (**d**) Comparison of the normalized pre-steady-state kinetics of USP4 CD and USP4 CD F386G ubiquitin dissociation. (**e**) Structure of the USP4 switching loop (SL) represented as yellow sticks (the rest of USP4 is coloured as in Fig. 1b). Residues mutated in **c** are labelled in red. Hydrogen bonds are indicated as black dotted lines. Blocking loop 2 (BL2) is shown with stick representation. Superposed ubiquitin is represented as a grey cartoon. (**f**) Schematic model representing the USP4 enzymatic turnover. The ubiquitinated target (T) is recognized by USP4 (1); ubiquitin hydrolysis is catalysed by USP4 (2); the deubiquitinated target dissociates from USP4 (3); ubiquitin dissociates from USP4 allowing the access of a new substrate (4). The dissociation of the DUSP–Ubl domain from the catalytic domain allows the switching loop (SL) to retain ubiquitin in the USP4 active site, preventing the access of more substrates (3a).

Since the DUSP domain is essential for USP4 activation, a series of mutants in the DUSP solvent-exposed residues (Supplementary Fig. 6A) were tested for activity on Ub–Rhod and 10 of these reduced USP4 FL activity (Fig. 5a,b). Many mutations (residues 88–92) affect the conformation of a loop of the DUSP domain that connects helix α4 and beta-strand β2 (Fig. 5a). One mutation is located at the N terminus of helix α2 (R40E) and two mutations (M24D and F51D) are located at the border of a deep hydrophobic cavity formed by helices α1, α2 and α5 (Fig. 5a). Finally, two mutations are located at the interface of the DUSP and Ubl domains (E134A and Y136A), suggesting that their relative orientation is important in USP4 activation.

The detrimental effect of Y136 mutation on activity was retained when DUSP–Ubl Y136A was added *in trans* (Supplementary Fig. 6B). However, when we measured the affinity of the mutant DUSP–Ubl for USP4 CD and insert by SPR we found that it is still able to bind to both USP4 constructs (Supplementary Fig. 6C), indicating that the destablization of DUSP–Ubl architecture does not interfere with its ability to interact with the insert but only with its role in promoting ubiquitin release by the USP4 catalytic domain.

### Mutations in the SL modulate USP4 activity

On the side of the catalytic domain, we found that mutation F386G in the SL region, near the catalytic site (Fig. 5e), substantially increases USP4 CD activity (Fig. 5c), in the absence of the DUSP–Ubl. Moreover, the initial ‘burst’ phase that characterizes USP4 CD is absent in this mutant (Supplementary Fig. 6D), suggesting that the enhanced activity is the result of enhanced ubiquitin off-rates and thus turnover. This was confirmed by the stopped-flow FP assay showing that USP4 CD F386G is able to release ubiquitin ~10-fold faster than the wild type (Fig. 5d; Table 2). Interestingly, the same mutation alters ubiquitin on-rates to a lesser extent (Supplementary Fig. 6E; Table 2), resulting in overall lower affinity for ubiquitin (Supplementary Fig. 6F).

In the full-length protein, the mutation F386G has the opposite effect, resulting in a decrease of activity compared with wild type (Fig. 5c; Table 2) and accordingly, ubiquitin off-rates are reduced in USP4 FL F386G relative to USP4 FL (Table 2). This activity remains higher than in USP4 CD F386G (Fig. 5c; Table 2), indicating that the DUSP–Ubl domain retains some ability to enhance catalytic turnover.

These opposing effects of this single F386G mutation argue that the SL is not only responsible for ubiquitin retention but rather provides a relay between the catalytic domain and DUSP–Ubl that promotes ubiquitin dissociation. This is confirmed by a second mutation (PQ385AV) in the SL (Fig. 5e). Whereas the activity of USP4 CD is not affected, the activity of USP4 FL is impaired (Fig. 5c), suggesting that the DUSP–Ubl domain targets P384 and/or Q385 to rearrange SL and favour ubiquitin dissociation. Interestingly, the SL loop is crucial also in the activation mechanism USP7 and Ubp8 (refs 8, 14, 15).

## Discussion

In this study, we show that the N-terminal DUSP–Ubl domain is required for the full enzymatic activity of USP4 *in vitro*. The catalytic turnover of USP4 catalytic domain is severely compromised by the strong retention of ubiquitin following substrate hydrolysis. Single-point mutants revealed that the SL in the ubiquitin tail-binding cleft plays a key role in the mechanism of ubiquitin retention. The DUSP domain promotes ubiquitin release by interfering with this mechanism, thus accelerating the catalytic turnover. The USP4 insert mediates the interaction between DUSP–Ubl and catalytic domain and a flexible connecting linker is required to allow sufficient conformational freedom for the two domains to interact.

The slow ubiquitin release by the USP4 catalytic domain is in contrast to the Michaelis–Menten assumption stating that the enzyme exists only in the free and substrate-bound forms and, accordingly, its enzymatic behaviour does not follow the model. A similar enzymatic behaviour, characterized by a ‘burst’ phase at the beginning of the reaction, has been described for other unrelated enzymes^39^,^40^. Enzyme kinetics have been analysed for a number of USPs and their isolated catalytic domains^30^,^41^,^42^, but, to our knowledge, this is the first example of slow ubiquitin release limiting the activity of a DUB.

The activation of otherwise largely inefficient catalytic domains has been reported for other USPs. USP7 catalytic domain is in a non-productive conformation and requires the C-terminal HUBL domain for activation^8^; similarly, Ubp8 alone is a catalytically dead enzyme and requires the allosteric activation by the other subunits of the SAGA complex^14^,^15^. The activation mechanisms described for these and other DUBs work by enhancing the affinity of the enzyme for the substrate, by making the catalytic step more efficient or by a combination of the two^8^,^14^,^15^,^41^. In this respect, the mechanism that we describe for USP4 is unique in that it acts on a different step of the enzymatic cycle, the release of the product. This unusual ‘allosteric regulation of product discharge’ may have a more general relevance in the modulation of DUBs and beyond, notably for other proteases.

USP4 inhibition *in vitro* occurs when the substrate is in excess relative to the enzyme (multiple turnover) to allow saturation by the product. On the other hand, when the enzyme is in excess, the activity of USP4 and its isolated catalytic domain will be dictated by the affinity for the substrate and the actual rate of the catalytic step. This, in combination with the presence of possible regulators, makes it difficult to predict how the described regulation mechanism affects the function of USP4 in the cellular environment.

Since the activation mechanism of USP4 relies on the DUSP–Ubl binding to the CD, a prominent question is whether USP4 is always in the activated state or whether the DUSP–Ubl domain alternates between the bound and unbound form (Fig. 5f), as it has been shown for the HUBL domain activating USP7 (ref. 8). In particular, recognition of ubiquitinated proteins may require the dissociation of the DUSP–Ubl domain as this may sterically interfere with the target by packing against the catalytic domain. The weak interaction between DUSP–Ubl and the catalytic domain (strongly impaired by point mutations) and the requirement of a flexible linker between the two argues that DUSP–Ubl has evolved to be able to dissociate from the catalytic domain. Interestingly, DUSP–Ubl has been implicated in the interaction with a substrate partner^18^, suggesting that the interplay between recruitment to the substrate and activation, as well as local ubiquitin concentrations, contribute to determine the outcome of USP4 action.

Single-residue mutations of the DUSP domain severely lower USP4 activity indicating that the DUSP domain is key for the activation of the enzyme. This is the first specific role described for a DUSP domain. Mutagenesis analysis also showed that the DUSP–Ubl tandem arrangement has functional implications, since mutations at their interface hamper USP4 activation. The structure of USP4 and USP15 DUSP–Ubl has shown some degree of flexibility between the two domains^25^,^26^, suggesting that the movement of the DUSP relative to the Ubl domain may be required to activate USP4. This would agree with a model where the Ubl domain anchors DUSP–Ubl to the catalytic domain by interacting with its insert (in agreement with the described scaffolding role of several Ubl domains^31^,^43^) and the DUSP domain dynamically interacts with the SL (Fig. 5f). Interestingly, the impairing mutations M24D and F51D are lying at the border of a deep hydrophobic cavity in the DUSP domain, which was suggested as a potential site for interactions, since it hosts the side chain of F127 belonging to a second DUSP–Ubl copy in the crystallographic asymmetric unit^26^.

This activation mechanism seems only partially conserved in the USP4 paralogues USP11 and USP15. Similar to the USP4 catalytic domain, USP11 FL is characterized by the initial ‘burst’ phase, indicating that its activity is limited by slow ubiquitin off-rates, but in this case the DUSP–Ubl domain is not able or not efficient in promoting ubiquitin release. In contrast, USP15 activity does not appear to be inhibited by the product, although the SL and the key residues for USP4 activation in the DUSP domain are conserved.

The critical function of USP4 in regulating pathways fundamental for cellular life is emerging, together with its involvement in cancer (notably its role in upregulating TGF-β response). The unique ‘allosteric regulation of product discharge’ that we describe contributes to the understanding of USP4 function at a molecular level, but may also have a broader impact in understanding the mechanisms behind the regulation of DUBs and other enzymes.

## Methods

### Plasmids and cloning

Complementary DNA for human USP4, USP15 and USP11 were a gift from Hidde Ploegh and Carlos Lopez Otin. USP4(296–954) and the D1 fragment (residues 296–490) of human USP4 were cloned using ligation-independent cloning into pET-46 Ek/LIC vector (Novagen). The D2 fragment (residues 766–932) and all other constructs were cloned into pET-NKI His-3C-LIC vectors^44^. USP4 Δinsert was created by inserting aa 353–359 of USP7 (SIKGKNN) between residues Leu479 and Leu777. USP4 mutants were created using the QuickChange mutagenesis kit (Agilent).

### Protein preparation

USP4, USP11 and USP15 variants were expressed in *E. coli* BL21(DE3) induced with 0.5 mM IPTG and grown at 20 °C overnight. For USP4 and USP15, cells were lysed in 20 mM Tris pH 7.5, 150 mM NaCl and 5 mM beta-mercaptoethanol or 1 mM TCEP. Proteins were purified by immobilized Ni^2+^ affinity chromatography, followed by anion exchange on a Resource Q column (GE Healthcare). After His-tag removal using 3C protease, the proteins were purified a second time on Ni^2+^ resin. The last purification step was size-exclusion chromatography (Superdex 200, GE Healthcare) in 20 mM HEPES pH 7.5, 150 mM NaCl and 2 mM dithiothreitol (DTT). USP4 D1D2 was coexpressed and purified according to the above scheme, without the anion-exchange step. For USP11, cells were lysed in 20 mM Tris pH 7.5, 500 mM NaCl, 10% glycerol and 5 mM beta-mercaptoethanol. The protein was purified similarly to USP4 and USP15 (without the anion-exchange step) keeping glycerol and 500 mM NaCl in the purification buffers.

### Limited proteolysis and protein identification

Purified USP4(296–954) (9 mg ml^−1^) was incubated with Thermolysin (0.8 units) for 1.5 h at room temperature and subjected to size-exclusion chromatography using Superdex 75 (GE Healthcare). Fractions containing USP4 D1 and D2 were subjected to liquid chromatography–mass spectrometry analysis. Liquid chromatography–mass spectrometry measurements were performed on a system equipped with a Waters 2795 Seperation Module (Alliance HT), Waters 2996 Photodiode Array Detector (190–750 nm), Waters Alltime C18 (2.1−100 mm, 3 μm), Waters Symmetry300TM C4 (2.1 × 100 mm, 3.5 μm) and LCTTM Orthogonal Acceleration Time of Flight Mass Spectrometer. Data processing was performed using Waters MassLynx Mass Spectrometry Software 4.1 (deconvolution with Maxent 1 function). N-terminal sequencing of USP4 D1 and D2 was performed by AltaBioscience (Birmingham, UK).

### Crystallization and structure determination of the USP4 D1D2

Crystals were grown overnight in sitting-drops mixing 200 nl USP4 D1D2 (~3.5 mg ml^−1^) with 200 nl 100 mM Bis-Tris propane pH 8.5, 25 mM Na_2_SO_4_ and 18% PEG3350 (w/v) at 19 °C. Crystals were cryoprotected in mother liquor and 25% ethyleneglycol. The crystals belong to the space group P2_1_2_1_2_1_ with six molecules per asymmetric unit (Table 1). Diffraction data were collected at the ESRF (Grenoble, France) beamline ID14-2 (wavelength 0.993 Å) and processed with MOSFLM^45^ and SCALA^46^. The structure was solved by molecular replacement with PHASER^47^ using USP8CD (PDB 2GFO) as a search model. Iterative rebuilding and refinement were done with Coot^48^, PHENIX^49^ and BUSTER^50^. The structure was validated with MOLPROBITY^51^ and WHAT-CHECK^52^ (Ramachandran statistics: preferred 1,923/ allowed 34/ outliers 0) and structure. Figures were generated using PYMOL^53^. Cysteine residue 311 in all chains have been chemically modified by beta-mercaptoethanol.

### SAXS analysis

Samples for SAXS measurements were prepared as described above. DTT (95 mM) was freshly supplemented before the measurements. Three to four sample concentrations were used ranging from 0.5 to 8 mg ml^−1^. Data were collected at ESRF beamline BM29 and analysed using the ATSAS software package^54^.

### Ub–Rhod activity assay

Activity on Ub–Rhodamine (gift from F. El Oualid and H. Ovaa) was measured with a PheraStar fluorescence plate reader (BMG) at 25 °C in 20 mM HEPES pH 7.5, 150 mM NaCl, 5 mM DTT and 0.05% Tween 20 in 384-well non-binding surface, flat bottom, low flange, black plates (Corning) and 30 μl final volume. Fifteen μl of Ub–Rhod was first manually added to wells, then 15 μl of enzyme was added either manually or with the injector of the plate reader. Fluorescence was measured at 1–30 s intervals at 485 and 520 nm excitation and emission wavelengths, respectively. For the Michaelis–Menten analysis the slope of the reaction initial linear phase was plotted as a function of substrate concentration [S] and fitted with the Michaelis–Menten equation *V*=(*V*_max_ · [S])/([S]+*K*_M_), using Prism 6 (GraphPad Software Inc.). *K*_cat_ was calculated as *V*_max_/[E], where [E] represents the enzyme concentration. All measurements were repeated at least three times.

### Ubiquitinated PCNA assay

^TAMRA−^ubiquitinated PCNA was prepared as described^34^ and mixed to USP4 CD and FL at the described concentrations. Aliquots of the reaction were loaded on SDS–polyacrylamide gel electrophoresis after being stopped with loading buffer at different time points. Bands corresponding to ^TAMRA−^ubiquitinated PCNA (S) and ^TAMRA−^ubiquitin (P) were quantified using ChemiDoc XSR (Bio-Rad). Product concentration was calculated as *S*_0_ · (P/(P+S)), where *S*_0_ is the initial substrate concentration.

### Stopped-flow pre-steady-state kinetics

Stopped-flow experiments were carried out in a TgK Scientific stopped-flow system (model SF-61DX2) equipped with a photomultiplier tube R10699 (Hamamatsu). A monochromatic light at 544 nm and a 570-nm cutoff filter were used for excitation and readout, respectively. The light was polarized using a calcite prism for the incident beam and dichroic sheet polarizers in front of each of two photomultiplier detectors arranged in a T-configuration. The experiments were performed at 20 °C in the same buffer used for Ub–Rhod activity assays; bovine serum albumin at 1 mg ml^−1^ was added to the buffer in some experiments to improve protein stability and did not affect the kinetics. In the dissociation experiments USP4 constructs at various concentrations (12.5–250 nM) were incubated with ^TAMRA−^Ubiquitin (2.5 nM final concentration) for 10–30 min and rapidly mixed with equal volumes of 2.5 μM of non-labelled ubiquitin (as a control, ubiquitin concentration was raised to 50 μM with no change in the kinetics). For the association experiments, equal volumes of USP4 (100–1,000 nM) and ^TAMRA−^Ubiquitin were rapidly mixed. Pre-steady-state dissociation kinetics data at different protein concentrations were globally fitted with a two-phase decay model 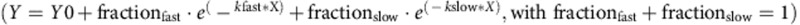; *k*_slow_, *k*_fast_ and their ratio were shared for all protein concentrations. The data could not be fitted with a single-exponential decay model. Association kinetics data were fitted to a single-exponential model 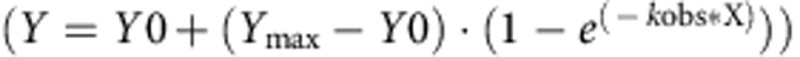. *K*_obs_ was plotted as a function of USP4 concentration and fitted to a linear equation and *k*_on_ calculated as the slope of the fitted line.

### FP equilibrium affinities

Equilibrium affinity experiments were carried out in the same setup as the Ub–Rhod assays. Different concentrations of USP4 were incubated with 2.5 nM ^TAMRA−^ubiquitin and FP measured with an excitation filter with a central wavelength of 531 nm, and P and S emission filters with a central wavelength of 579 nm. The FP values were plotted as a function of concentration ad fitted to a one-site binding model accounting for ligand depletion using Prism6 (GraphPad Inc.)

### SPR measurements

USP4 CD, insert and D1D2 were biotinylated on lysines using half molar amounts of EZ-Link Sulfo-NHS-LC-LC-biotin (Thermo Scientific). SPR was performed at 25 °C on a Biacore T100 (GE Healthcare) using a SA sensor chip loaded with ~100 response units of biotinylated USP4. Concentration series of USP4 DUSP–Ubl in running buffer (20 mM HEPES pH 7.5, 150 mM NaCl, 5 mM DTT and 0.05% Tween 20) supplemented with NSB reducer (GE Healthcare) were flowed over the chip at 30 μl min^−1^. The program EvilFit was used to calculate populations of dissociation constants (*K*_d_) with similar values for dissociation rates (*k*_off_) allowing to remove the contribution of nonspecific accumulation (slow *k*_off_ component) to the overall dissociation constant.

## Author contributions

M.C. designed, carried out and analysed all experiments, with the exception of D1D2 identification and crystal structure (M.P.A.L.-V. with assistance from A.C.F.). The project was supervised by T.K.S. M.C. and T.K.S. wrote the manuscript with comments from all authors.

## Additional information

**How to cite this article**: Clerici, M. *et al.* The DUSP–Ubl domain of USP4 enhances its catalytic efficiency by promoting ubiquitin exchange. *Nat. Commun.* 5:5399 doi: 10.1038/ncomms6399 (2014).

**Accession codes:** Atomic coordinates and structure factors have been deposited in the Protein Data Bank under accession code 2Y6E.

## Supplementary Material

Supplementary Information Supplementary Figures 1-6.

## Figures and Tables

**Table 1:** Data collection and refinement statistics.

	**USP4 D1D2**
*Data collection*	
Space group	P2_1_2_1_2_1_
Cell dimensions	
*a*, *b*, *c* (Å)	110.50, 151.03, 178.67
*α, β, γ* (°)	90.0, 90.0, 90.0
Resolution (Å)	44.6–2.4 (2.53–2.4)
*R*_merge_ (%)	8.8 (66.1)
*I*/*σI*	11.0 (1.1)
Completeness (%)	94.9 (74.0)
Redundancy	3.4 (2.4)
*Refinement*	
Resolution (Å)	44.6–2.4 (2.53–2.4)
No. of reflections	111,095
*R*_work_/*R*_free_	17.8/21.0
No. of atoms	
Protein	15,884
Ligand/ion	8
Water	1,249
*B*-factors	
Protein	52.52
Ligand/ion*	116.7
Water	49.65
R.m.s.d.	
Bond lengths (Å)	0.010
Bond angles (°)	1.02

USP, ubiquitin-specific protease.

^*^Six Zn^2+^ and two SO_4_^−^ ions.

**Table 2:** Enzymatic and kinetic parameters of USP4 and USP15 constructs

	***k***_**cat**_ **(s**^−**1**^**)**	***K***_**M**_ **(**μ**M)**	***k***_**slow**_ **(s**^−**1**^**)**	***k***_**fast**_ **(s**^−**1**^**)**	***k***_**on**_ **(μM**^−**1**^ **s**^−**1**^**)**	***K***_**D**_ **(μM)**
USP4 FL isof. 1	0.30±0.07	0.15±0.05	0.053±0.003 (36%)	0.55±0.016 (64%)	5.1±0.23	0.092±0.021
USP4 CD			0.0013±0.00001 (90%)	0.011±0.0008 (10%)	0.074±0.017	0.044±0.007
USP4 FL isof. 2	0.29±0.06	0.20±0.05				
USP4 FL Δinsert	0.042±0.002	0.16±0.09				
USP4 FL F386G	0.13±0.02	0.25±0.03	0.024±0.002 (30%)	0.26±0.010 (70%)		
USP4 CD F386G	0.027±0.002	0.16±0.04	0.015±0.0006 (79%)	0.12±0.016 (21%)	0.035±0.007	~1
USP4 FL PQ385AV	0.092±0.007	0.14±0.01				
USP4 D1D2	0.030±0.004	0.086±0.030				
USP15 FL	0.15±0.004	0.20±0.03				
USP15 CD	0.05±0.004	0.10±0.02				

USP, ubiquitin-specific protease.
