# Supplementary material for: The DUSP–Ubl domain of USP4 enhances its catalytic efficiency by promoting ubiquitin exchange
Source: Nat Commun. 2014 Nov 18;5:5399. doi: 10.1038/ncomms6399 (PMC4243247; doi:10.1038/ncomms6399)
Supplement: Supplementary Information — Supplementary Figures 1-6. [file ncomms6399-s1.pdf]

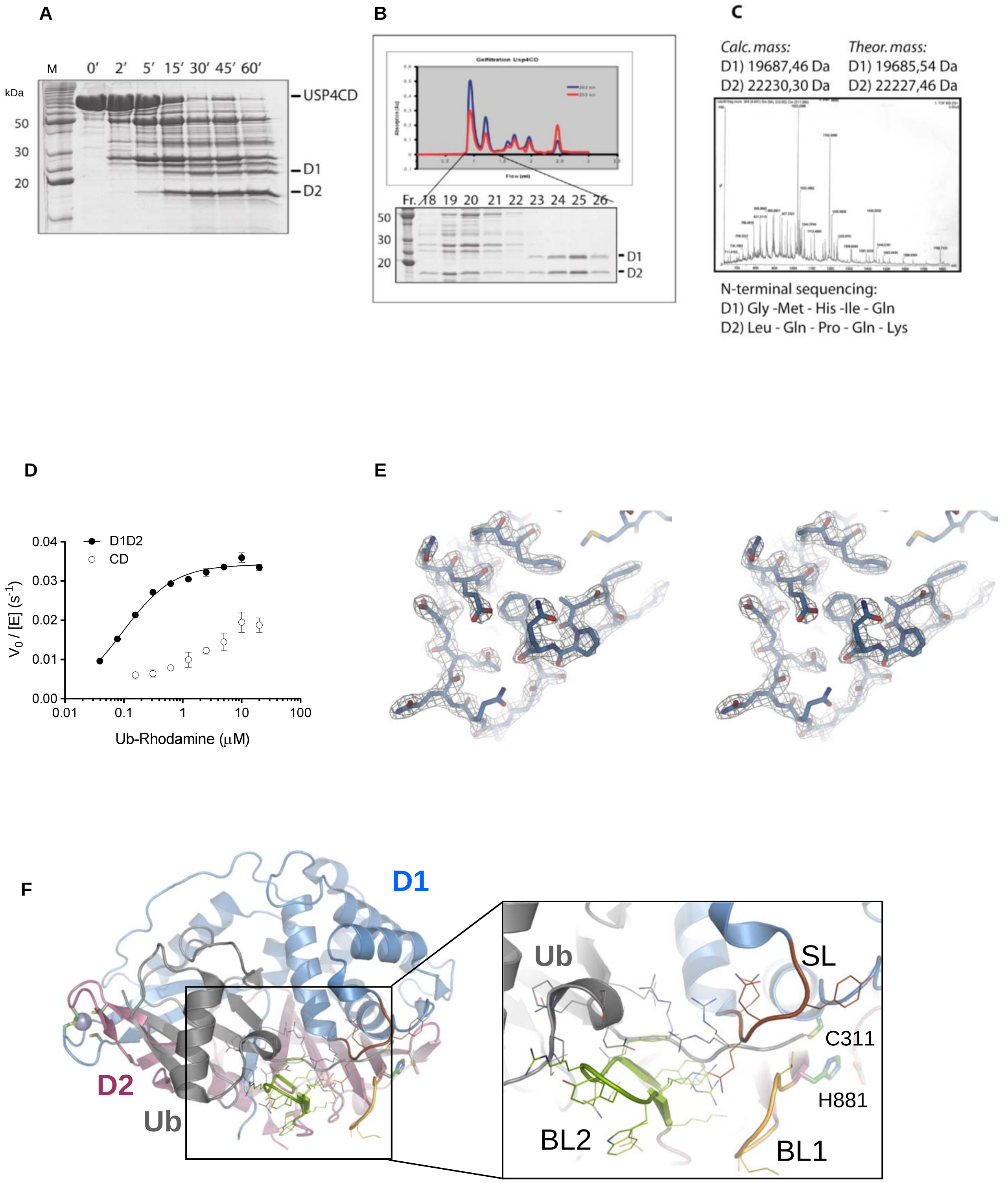

Supplementary figure 1. **Identification of USP4-D1D2.** (**A**) Limited proteolysis analysis of thermolysin cleavage on USP4(296-954) at 37°C. The reaction was stopped at different time points and samples analyzed on SDS-PAGE gel. (**B**) Size exclusion chromatography of a proteolytic sample of USP4(296-954). Fraction samples were analyzed on SDS-PAGE gel. (**C**) Mass spectrometry analysis and N-terminal sequencing results for the D1 and D2 fragments. **(D)** Michaelis-Menten plot showing the catalytic rate of USP4 D1D2 and CD as a function of substrate concentration on the minimal substrate ubiquitin-rhodamine. **(E)** Stereo image of a portion of the 2Fo-Fc electron density map (2.0 σ) **(F)** Model of USP4 D1D2 in complex with ubiquitin showing that the position of BL1 (green), BL2 (orange) and BL3 (brown) loops is incompatible with ubiquitin (grey) access and release from the catalytic domain. Ubiquitin coordinates were taken from the USP2-ubiquitin complex superposed to USP4 D1D2. **(G)** *next page*.

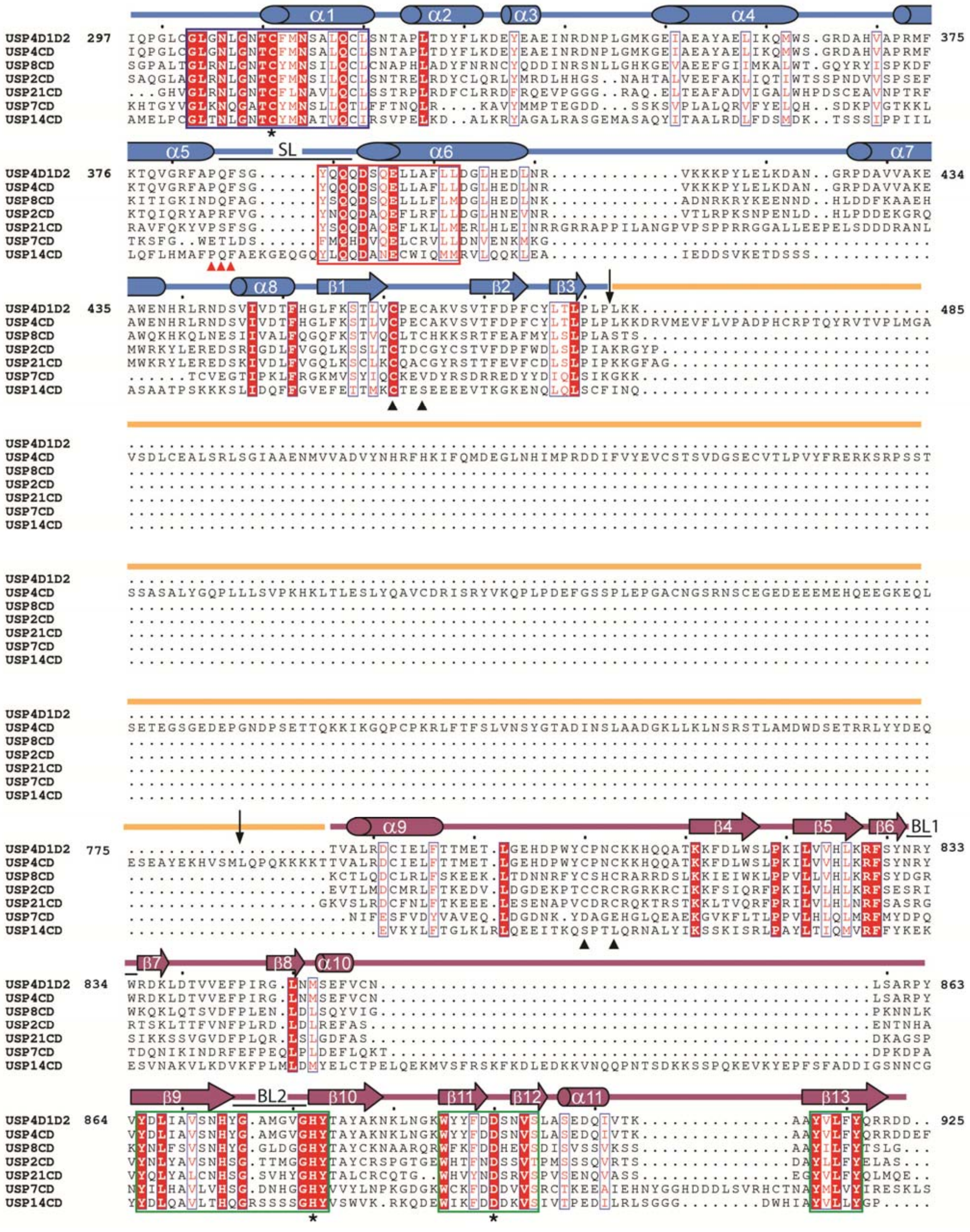

Supplementary figure 1 *(continued)* **(G) Structure-based multiple sequence alignment.** Secondary structure elements are colored and labeled according to Figure 1. The two black arrows indicate the thermolysin cleavage sites in USP4. The catalytic triad residues are indicated with an asterisk; the four Cys residues coordinating the zinc ion are indicated with black triangles, mutations altering USP4 activity are indicated with red triangles. Cys, QQD and His boxes are indicated in blue, red and green, respectively.

A

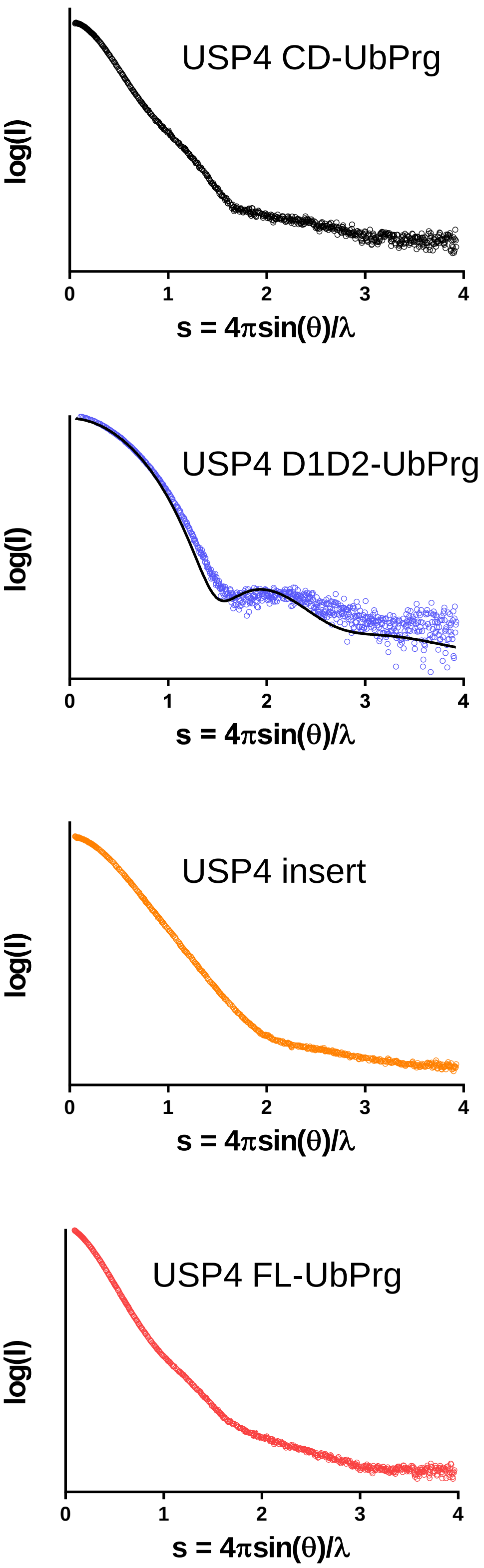

B

	MW [kDa]	Rg [Å]	D_{max} [Å]
USP4 CD-UbPrg	82.0 (80.2)	43.9	133
USP4 FL-UbPrg	117.3 (112.6)	46.9	145
USP4 D1D2-UbPrg	51.1 (50.5)	25.9 (22.9)	75 (70)
USP4 insert	34.3 (31.7)	30.6	90

C

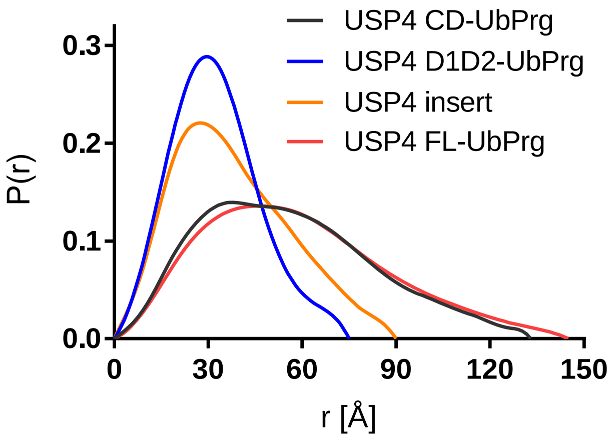

D

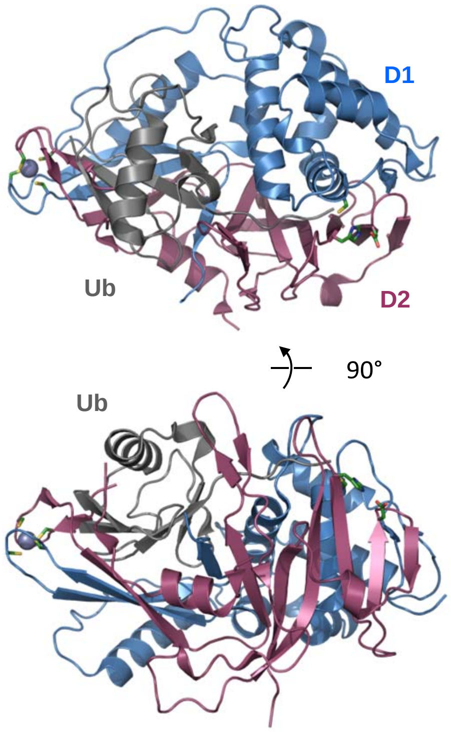

Supplementary figure 2. **Small Angle X-ray Scattering analysis of USP4 catalytic domain.** (**A**) Scattering curves of USP4 CD and D1D2 in complex with UbPrg and USP4 insert and calculated scattering curve (black line) for the D1D2-UbPrg complex model crystal structure. (**B**) Calculated parameters for the SAXS experiments (Expected values are indicated in brackets). (**C**) Interatomic distance probability distribution (P(r)) for the USP4 constructs. **(D)** Front and side view of the model of USP4 D1D2 in complex with ubiquitin. Ubiquitin coordinates were taken from the USP2-ubiquitin complex superposed to USP4 D1D2 subunit A.

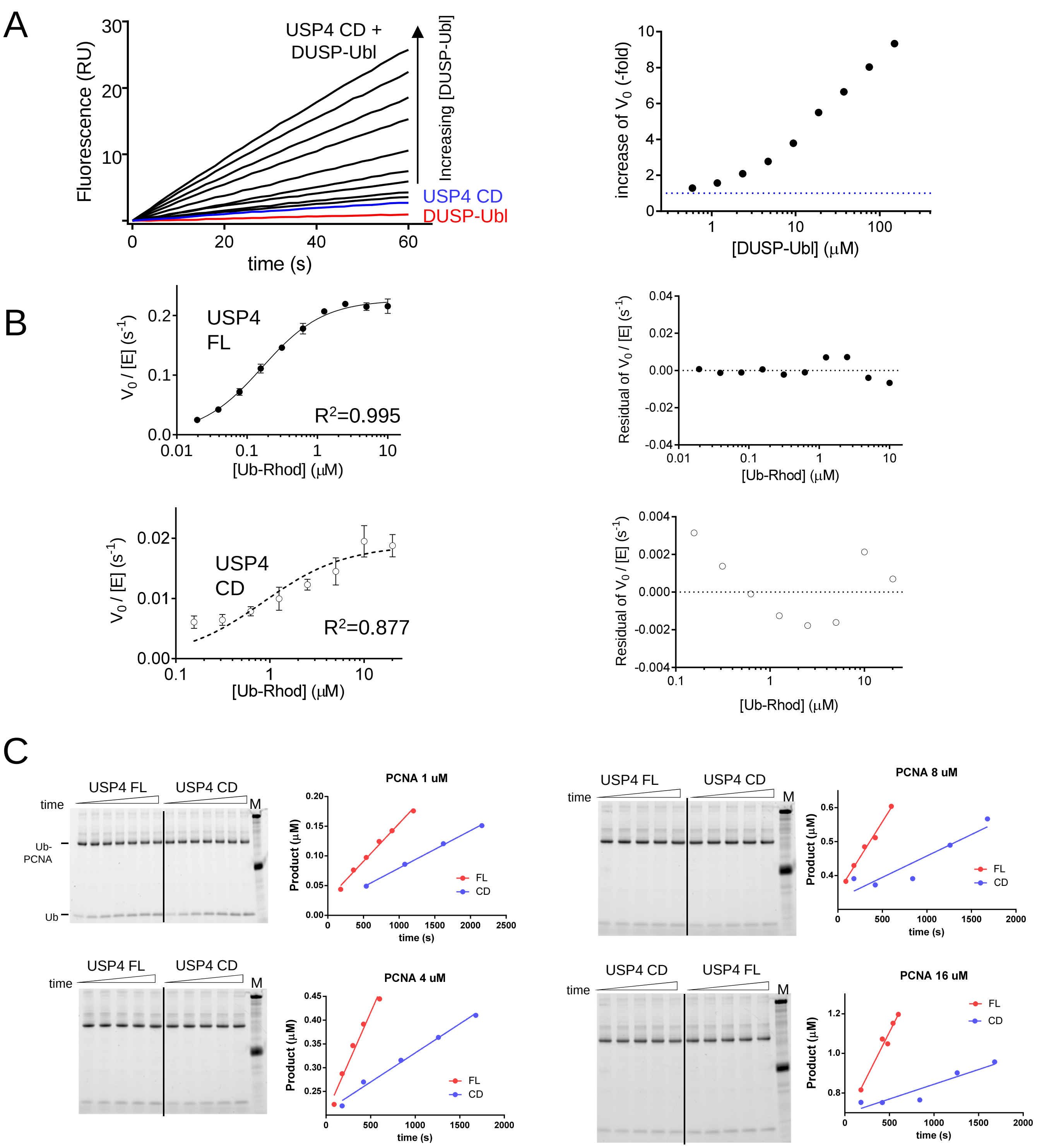

Supplementary figure 3. (**A**) Time course plot of USP4 DUSP-Ubl in *trans* activity assay on Ub-Rhod (left panel). USP4 CD (50 nM) was mixed with increasing concentrations of DUSP-Ubl (0.6, 1.2, 2.3, 4.7, 9.4, 18.8, 37.5, 75 and 150 μM) and used to hydrolyze 5 μM of Ub-Rhod substrate. As a control, USP4 CD alone was used (blue curve). DUSP-Ubl alone at 600 μM was also used as control and showed no significant activity (red curve). Initial reaction rates normalized to the reaction rate of USP4 CD alone are plotted as a function of the added DUSP-Ubl concentration (right panel). **(B)** Catalytic rate vs substrate concentration plots fitted with a Michaelis-Menten model for USP4 CD and FL (left panels). Residuals (difference between experimental data and model) for the Michaelis-Menten fitting (right panels). **(C)** PCNA deubiquitination gel assays. USP4 FL and CD (20nM) were incubated with $^{TAMRA-}$Ub-PCNA at the indicated concentrations and the reaction stopped at different time points and loaded on SDS-PAGE. The amount of product was quantified and plotted as a function of time. The initial reaction rates (slopes) for every substrate concentration were calculated and the average of three repetitions were plotted as a function of PCNA concentration in figure 2A.

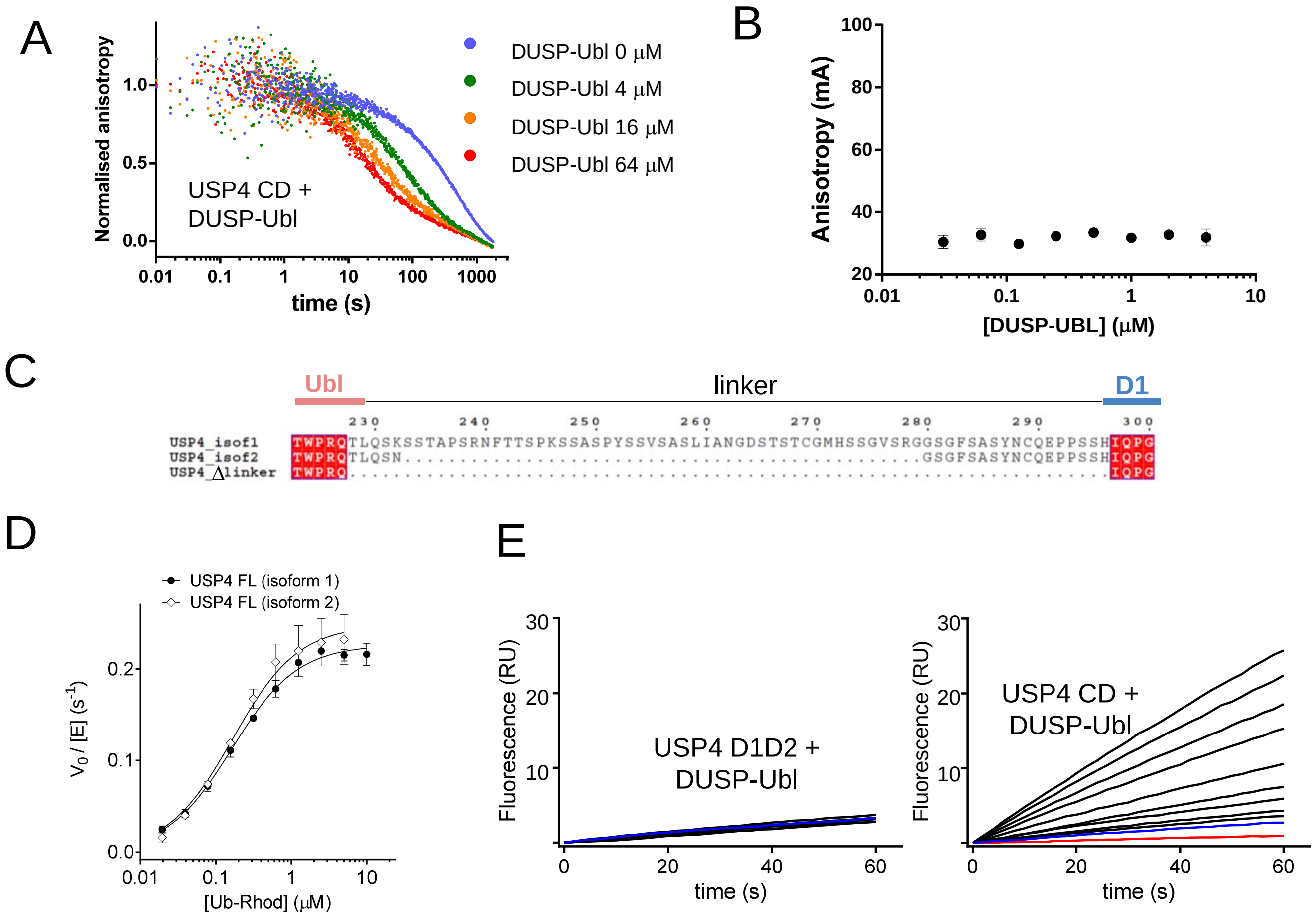

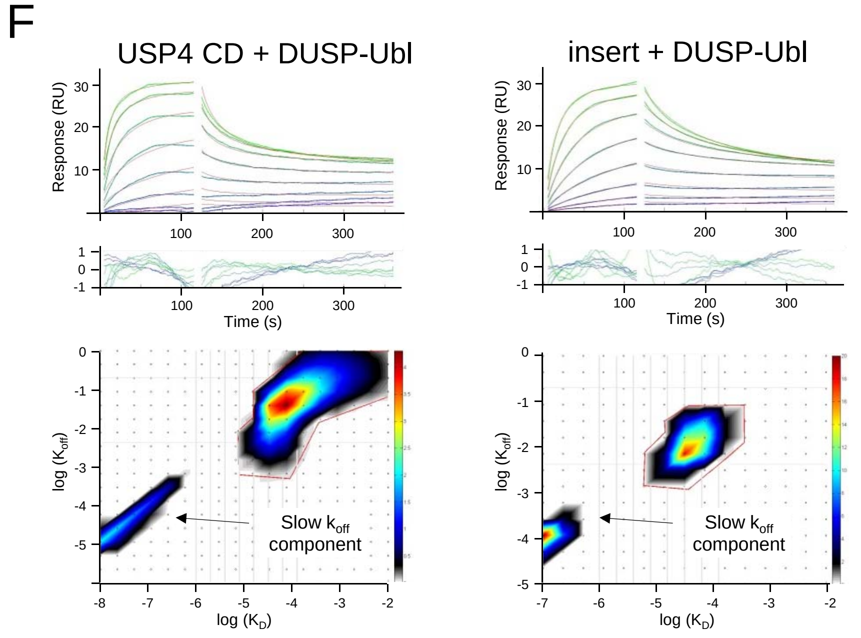

		DUSP-Ubl	DUSP-Ubl Y136A
**USP4 CD**	K_{Dfit} (μM)	109 ± 49	81 ± 53
**USP4 insert**	K_{Dfit} (μM)	44 ± 26	31 ± 4

Supplementary figure 4. (A) Stopped-flow fluorescence polarisation (FP) measurements of ubiquitin disassociation from USP4 CD at increasing concentrations of DUSP-Ubl added *in trans*. **(B)** Equilibrium FP assay of DUSP-Ubl binding to ubiquitin.(**C**) USP4 Δlinker, isoform 1 and 2 linker sequence (**D**) Michaelis-Menten plots for USP4 FL isoform 1 and 2. The relative catalytic parameters are listed in table 2. **(E)** Activity assay DUSP-Ubl addition in *trans* to D1D2 (left panel). Addition to USP4 CD is shown for comparison (right panel). **(F)** Example of fitted USP4 CD and insert SPR binding kinetics of DUSP-Ubl using EvilFit (Svitel *et al*, 2003; Svitel *et al*, 2007). Data is represented in a gradient of green to blue for multiple protein concentrations; red lines indicate fitted model curves. Residuals of the fits are plotted below the graph (top). Heat map of k_{off} and K_D distribution (bottom). A red line is drawn around the area for which a weighted average was taken for determination of the kinetic parameters. Apparent dissociation constants (K_{Dapp}) and calculated with EvilFit (K_{Dfit}) are shown (right).

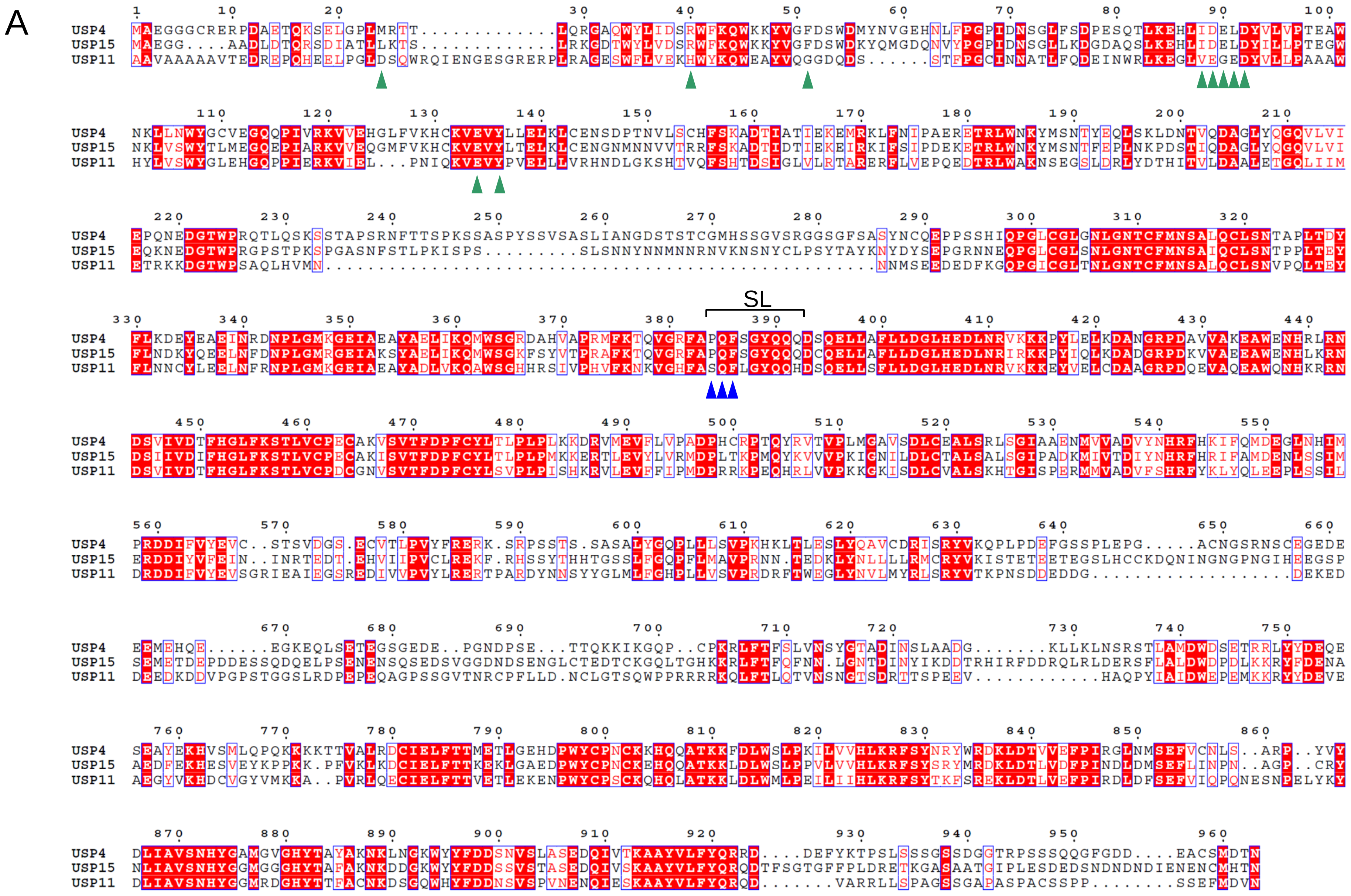

Supplementary figure 5. *(Legend in the next page)*

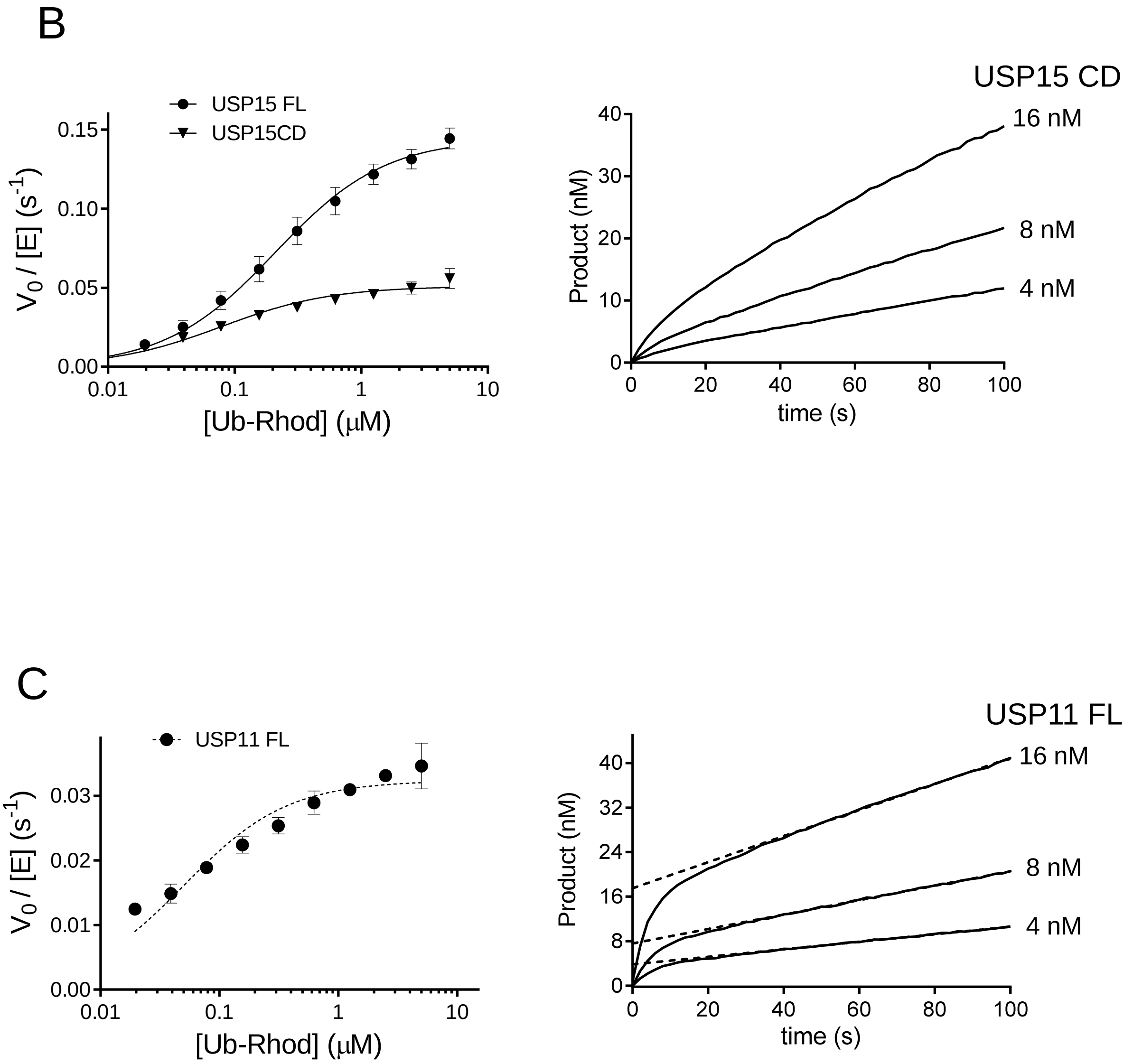

Supplementary figure 5. **Kinetic behaviour of USP4 paralogs USP15 and USP11. (A)** USP4, USP15 and USP11 sequence alignment (*previous page*). DUSP and SL residues important for USP4 activity are indicated by green and blue triangles, respectively. **(B) (C)** Michaelis-Menten plot (left panel) and reactions at 160 nM Ub-Rhod and three different enzyme concentrations (right panel) for USP15 (B) and USP11 (C).

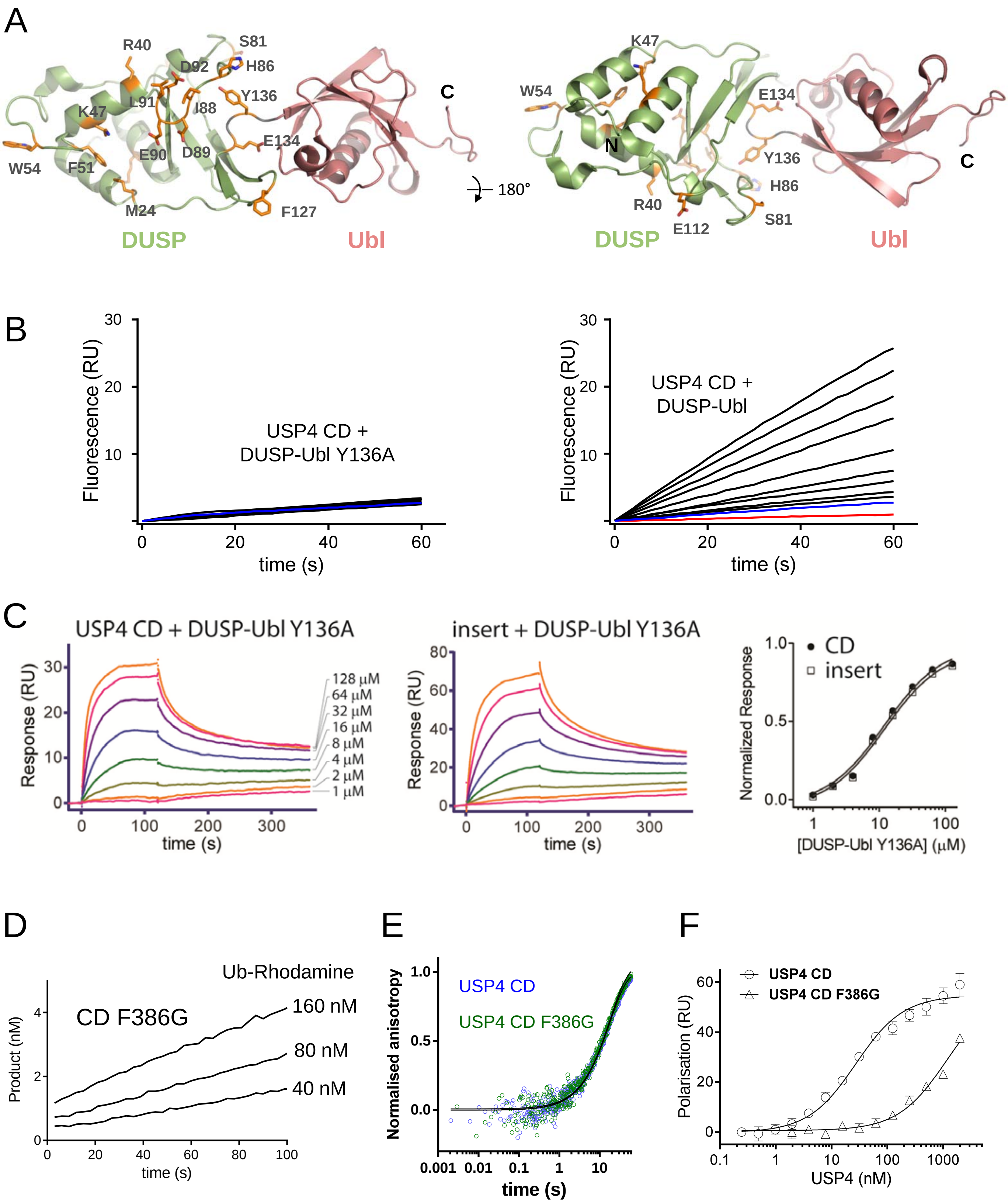

Supplementary figure 6. (**A**) Location of all residues mutated in the DUSP-Ubl domain (represented as orange sticks) **(B)** USP4 DUSP-Ubl in *trans* activity assay (as in supplementary figure 6A). DUSP-Ubl Y136A loses the ability to enhance USP4CD activity (left panel); wild type DUSP-Ubl is shown for comparison (right panel). **(C)** SPR binding studies of DUSP-Ubl Y136A interacting with USP4 CD and insert (left and middle panels). Overlay of normalised equilibrium responses (right panel). (**D**) USP4 CD F386G reactions at different Ub-Rhod concentrations and 3 nM enzyme. **(E)** Stopped flow on-rates of USP4 CD F386G (green) compared to wild type USP4 CD (blue). **(F)** Ubiquitin equilibrium affinities of USP4 CD and CD F386G measured with FP.
